# Weight reduction after bariatric surgery - Do results correlate with postoperative visit frequency?

**DOI:** 10.1016/j.obpill.2023.100093

**Published:** 2023-11-05

**Authors:** Anila Punjwani, Gabrielle Unbehaun, Zachary Duarte, Juaquito Jorge

**Affiliations:** a Saint James School of Medicine - Anguilla, A-1 Albert Lake Dr, The Quarter 2640, Anguilla; b New Hope Surgical/Suburban Surgery Center, 1950 N Harlem Ave., Elmwood Park, IL 60707, USA; c St. George's University School of Medicine, University Centre, Granada, West Indies

**Keywords:** Bariatric surgery, Medical weight reduction, Surgical weight reduction

## Abstract

**Background:**

Consistent follow up after a bariatric surgery is considered vital to adequate and sustained weight reduction. This study examined the strength to which compliance with postsurgical appointments predicted an increase in total body weight loss percent (TBWL%) and change in body mass index (BMI) in a cohort of Illinois based surgical center patients.

**Methods:**

Participants (n = 78) were patients with obesity who underwent bariatric surgery between 2019 and 2021. Patient preoperative weight and BMI, and postoperative weight and BMI at 3-month intervals for one year, and the number of visits were collected from chart review to calculate TBWL%, and change in BMI. Relationship between these factors and postoperative visit compliance were evaluated using linear regression to assess if it was a significant predictor of success.

**Results:**

Follow up visit compliance had a weak positive association with increased TBWL% (Model R^2^ = 0.134, B = 0.011, p = 0.004) and a minimally stronger association with increased change in BMI (Model R^2^ = 0.229, B = 0.015, p = 0.0001).

**Conclusions:**

Adherence with follow up visits was weakly related to improved weight reduction outcomes after bariatric surgery. Future investigations should consider how follow up visits can better influence a patient's success in sustaining achieved weight reduction.

## 1 Introduction

Obesity is a chronic disease that affects more than 30% of the world's population. Since 2000, the prevalence of severe obesity has nearly doubled, requiring physicians to incorporate more obesity management into their practice [1]. Like any other chronic disease, the illness cycles through a pattern of diagnosis-treatment-remission-recurrence. Individuals with a body mass index (BMI) that is categorized as overweight or obese are at an increased risk for a variety of mental and physical illnesses, which include but are not limited to anxiety, depression, diabetes type II, dyslipidemia, gallbladder disease, heart disease, hypertension, osteoarthritis, sleep apnea, and stroke [2].

First-line treatment for patients with obesity includes behavioral therapy, lifestyle modifications, and treatment for underlying conditions predisposing the patient to weight gain. While important, these methods are often unsuccessful for the patients who would benefit the most from significant weight reduction. Additionally, these behavioral and dietary changes often result in only a 5–10% reduction in total body weight with a weight recurrence rate near 80% weight recurrence within five years [3]. Pharmaceutical weight reduction options include anti-obesity medications, medications targeting the hormonal pathways for satiety, and antihyperglycemic agents [4]. In addition, physicians can consider the variety of bariatric surgeries as options for significant and sustainable weight reduction in patients who have failed other approaches. Though bariatric surgeries exist on a spectrum, most work through a combination of gastric size restriction, decreased intestinal absorption, and alteration of the neurohormonal pathway. Selecting the appropriate bariatric surgery should be a shared decision between the patient and physician, with a comprehensive review of the patient's health history, desires, and potential complications of each option. The presence and severity of comorbid metabolic conditions can provide further insight into which procedure would best benefit the patient. For example, while sleeve gastrectomy and gastric bypass are both effective in putting diabetes mellitus into remission the biliopancreatic diversion with duodenal switch has the highest overall rate of remission. Gastric bypass surgery diverts from the hypoabsorptive limb, increases GLP-1 production, increases insulin secretion and sensitivity, reduces appetite by decreasing ghrelin and leptin, and increases satiety through peptide YY. Selecting any bariatric surgery should be a multifaceted decision and patients should be given adequate time to consider all possibilities. Factors should include previous attempts to lose weight over the last 2- years, medical conditions contributing to weight gain, comorbidities such as diabetes mellitus, hypertension, hyperlipidemia, and conditions that might be contraindications to surgery. In addition, patients should consider personal habits, risk aversion, and lifestyle when selecting a surgical approach. Lastly, patients should strongly consider the potential complications of any surgical procedure and the consequences of each. Overall, bariatric surgery is a tool to help patients in their weight reduction journey. Regardless of which surgery is selected, patient effort with diet, exercise, follow-up appointments, and support groups will ultimately determine their success in meeting and maintaining their weight reduction goals. Preoperative requirements for bariatric surgery vary by procedure, provider, and insurance but often include a minimum of one psychosocial evaluation, more if clinically merited based on patient history, two to six diet and nutrition classes, and preoperative health clearance including esophagogastroduodenoscopy, colonoscopy (if over age 45), general lab workup and obstructive sleep apnea assessment. The patients are provided resources for counseling, support sessions, and nutrition education [5,6]. Postoperative expectations can vary between providers but generally include a one-week follow-up visit to assess wound healing and discuss early dietary restrictions and regular monthly follow-up visits for the first year. Patients should adhere to a clear liquid diet for the first 2-weeks postoperatively, a full-liquid diet for an additional 2-weeks, then a pureed diet for the next 4-weeks before progressing to an adaptive stabilized diet that they should maintain for life. In addition to regular follow-up visits with a health care provider, patients should consider nutrition and diet classes and bariatric support groups. These support sessions help patients implement and maintain healthy eating habits [7]. As hypothesized, the relationship between increased postoperative follow-up visits is related to an increase in the TBWL%. As a secondary hypothesis, we investigated the relationship between postoperative visit frequency and the change in BMI.

## 2 Research methodology

### 2.1 Study design

A retrospective analysis was conducted using the surgical database established and maintained at New Hope Surgical Center at a community-based hospital in Illinois. Data was retrieved for height, weight at pre-operative appointment, and postoperative weight at 3-month intervals until 1 year, and the number of follow-up visits attended with the practice. Data was included from patients who underwent bariatric surgery from 2019 to 2021, had at least 2 data points after surgery, and did not start any pharmaceutical weight loss in the first year after surgery. Patients who commenced pharmaceutical weight loss were excluded to prevent confounding.

### 2.2 Participants

The sample consisted of adult patients (≥18 years) with obesity who underwent bariatric surgery by the primary surgeon between 2019 and 2021. Patients need to have participated in their postoperative follow-up appointments during the first year. Patients who underwent bariatric surgery without attending follow-up appointments and those who participated in medication-assisted weight reduction were excluded from this study. The number of bariatric surgeries during the study limited the sample size. A total of 60 patients met this criterion which represents approximately 77 % of the total bariatric surgeries performed during the specified time frame.

### 2.3 Primary outcomes

Patients that met, or surpassed, the minimum visit requirement of 12 visits within one year postoperatively noticed a greater decrease in their overall weight. Twenty-eight percent of patients had a >30 % TBWL% and 42 % of patients had a >30 % change in BMI.

### 2.4 Intervention

We reviewed patient records to see how many visits they attended in the one-year postoperative period and how it correlated to a decrease in BMI and an increase in TBWL%.

### 2.5 Diagnostic assessment

BMI calculation used: weight (lb)/[height (in)]2 × 703 weight and height values were obtained as recorded within the EMR at the patient's initial visit and each subsequent follow-up visit. Their pre and postoperative clinical data and anthropometric measurements were collected and recorded during follow-up, providing us with target weight recordings in 3-month intervals for at least one year postoperatively.

### 2.6 Statistics

This study is designed to assess the hypothesis that patients who have an increased number of postoperative follow-up visits will have an increased total body weight loss (TBWL)% and a greater overall decrease in BMI. To compare patients with a variety of initial weights, patients were compared using the percent of total body weight loss and percent change in BMI. Initial weights and height were collected during the preoperative visit and subsequent weights were collected during bariatric follow-up visits. Weight entries were divided into 3-month periods, with only the lowest weight for that period reported. Total body weight loss was found by taking the total weight lost during the one-year postoperative period and dividing this number by the pre-surgery weight of the patient. The number of follow-up visits and the number of dietary classes attended were determined by the electronic health record. The preoperative and postoperative BMIs of the patients were calculated from data in the electronic health records. Linear regression was then performed using Excel (Version 16.69.1 published by Microsoft). A p-value of <0.005 was used to determine statistical significance.

## 3 Results

From January 1, 2019 to December 31, 2021 bariatric surgery was performed on 78 patients by one surgeon. We excluded 11 cases who commenced with medical weight reduction within the first year of their surgery. An additional 7 cases were excluded from the study as the patients were lost to follow-up before the timeline of the study concluded. Among the remaining 60 patients, 40 patients (67 %) underwent SG, 17 patients (28 %) underwent gastric bypass, 2 patients (3 %) underwent laparoscopic adjustable gastric band, and 1 patient (1.6 %) underwent stomach intestinal pylorus-sparing surgery. The mean number of follow-up visits in 1 year was 5.6 (median 4.5, range 1–17). The majority of the patients were female (95 %, n = 57).

**Fig. 1:**
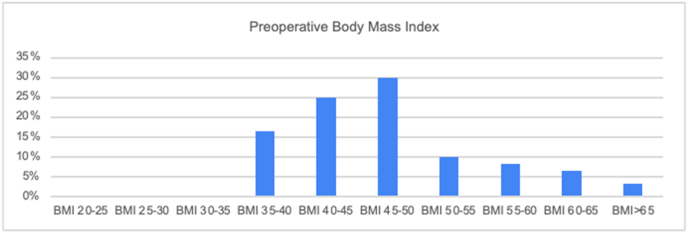
Stratification of included patients in the study and their pre-operative body mass index values.

**Fig. 2:**
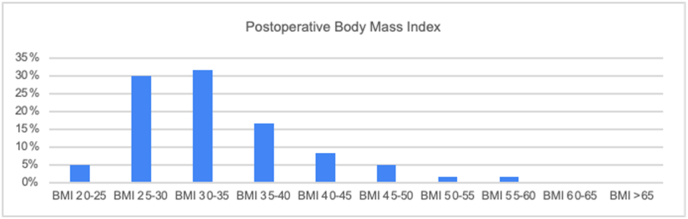
Stratification of included patients in the study and their post-operative body mass index values.

The average preoperative BMI of participants was 47.34 kg/m2 (median 45.76, range- 35.27 to 70.04) (Fig. 1). Of the 60 patients included in the study, the overall mean total body weight loss over 1 year was 25.36 % (median 25.11 %, range: 2.72–50.69 %). Ninety-five percent of patients had TBWL >10 % (n = 58), 66 % obtained >20 % (n = 40), and 28 % obtained >30 % (n = 17). The average postoperative BMI of participants one year after surgery was 33.62 kg/m2 (median 31.88, range: 20.83 to 55.57) (Fig. 2). The average change in BMI of participants was 28.7 % (median 27.3 %, range: 6.5 to 54.1). Ninety-eight percent of patients had a >10 % (n = 59) decrease in BMI, 76 % had a >20 % (n = 46) decrease, 42 % had >30 % (n = 25) decrease in BMI and 13 % had >40 % (n = 8) decrease in BMI. The average excess body weight lost was 56.66 % (median 60.71 %, range: 4.69 %–112.76 %).

**Fig. 3:**
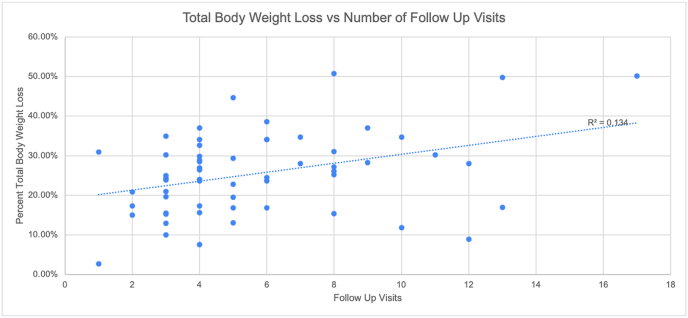
An increase in the number of follow up visits is weakly positively correlated (R = 0.134) with an increase in Total body weight loss percent (TBWL%).

**Fig. 4:**
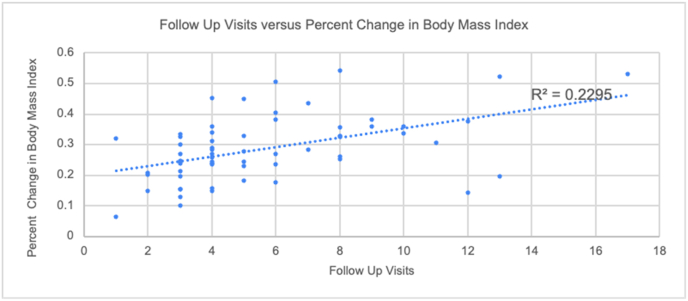
An increase in the number of follow up visits is positively correlated (R = 0.2295) with a change/decrease in body mass index.

We performed a linear regression analysis to assess the predictive factor of the number of follow-up appointments and the number of diet classes on the percent total body weight loss and the change in BMI hat one year after surgery. When comparing the relationship between follow-up appointment and the TBWL%, the resulting linear model fits the data with an R2 value of 0.134 with B = 0.011 (Std. error = 0.003, 95%CI: 0.008–0.014, p = 0.004) (Fig. 3). When comparing the relationship between the number of follow-up appointments and the percent change in BMI, the resulting linear model had an R2 value of 0.229 with B = 0.015 (Std. error = 0.0037, 95%CI: 0.011–0.019, p = 0.0001) (Fig. 4). The relationship between the number of dietary classes and %TBWL was shown to have an insignificant negative relationship with an R2 value of 0.017 with B = −0.005 (Std. error 0.005, P-value = 0.324).

## 4 Discussion

### 4.1 Implications

We need to address and account for confounding variables during follow-up appointments. Providers should not rely exclusively on BMI when assessing the progress of their patients as BMI does not take into account muscle mass, bone density, and overall body composition. A person with more muscle will weigh more than someone with less muscle at the same height and therefore will have a higher BMI despite being arguably more healthy. Follow-up appointments do not negatively correlate with TBWL% or decrease in BMI, therefore while they do not guarantee significantly different results, with their positive correlations they support the potential. Furthermore, follow-up visits are what the patient and provider make of them; whether they choose to accept and put into practice the guidance given by the provider [8]. Providers should be mindful to provide meaningful, evidenced-based behavior modification recommendations supported by motivational interviewing.

### 4.2 Factors that may be influencing a weak association

While patients may attend more follow-up visits, they may not be adhering to proper diet guidelines as outlined in their nutrition counseling session. Factors that may affect adherence to a proper diet include but are not limited to cravings, financial issues, lack of cooking experience and knowledge, the convenience of unhealthy food, and dietary restrictions, sensitivities, and preferences. To combat this, patients are encouraged to continue to attend nutrition and diet classes, seek out a dietician as needed, and continue to attend follow-up appointments to monitor progress. Center staff are readily available to help patients set up meal plans and exercise routines that consider the patient's preferences. Patients may also struggle with post-surgery depression, lack of motivation to exercise, or life factors preventing them from exercising. For lack of motivation, patients are encouraged to continue to attend support groups, whether online or in person through the practice, so they have someone they can relate to. Advice offered to patients includes making a friend going through the same situation which can help with accountability and motivation. Weight reduction and weight reduction maintenance is fostered with patient encouragement through meaningful, evidenced-based behavior modification recommendations supported by motivational interviewing. Pre and postoperative visits should assess patients for weight reduction maintenance barriers. Weight reduction maintenance barriers such as, impaired sleep, impaired ambulation, self-body perception, mental stress, depression, food cravings, bias, discrimination, employment issues can contribute to difficulty in achieving weight reduction and lead to weight recurrence. The patient provider partnership should further focus on how to eliminate or overcome these barriers to achieve weight reduction goals.

Patients are asked to keep their expectations realistic and not expect results to happen more quickly, which may be discouraging when expectations are not met. There are many systems in place to help the patient achieve their weight reduction goals and prevent weight recurrence.

### 4.3 Limitations

In addition to these confounding factors, the sample size of our population was small and this could influence the strength of the association. Further investigations with a larger sample size would illuminate further the strength of the relationship. In addition, future research should investigate methods to increase follow-up visits and ensure active attendance to these appointments.

## 5 Conclusion

This study looked at patients who underwent various bariatric surgeries and the relationship between the number of follow-up visits with their BMI and TBWL%. We hypothesized that patients who have an increased number of postoperative follow-up visits will have an increased TBWL% and increased change in BMI. Our hypothesis was mildly supported. After adjusting for patients who initiated medical weight reduction within one year postoperatively and patients who were lost to follow-up before their initial one-year postoperative period ended, there was only a weak positive correlation between follow-up visits and increasing TBWL% change. However, there was a slightly stronger association between follow-up visits and overall change in BMI. Future investigations should consider how follow up visits can better influence a patient's success in sustaining achieved weight reduction.

## Author contribution (CRediT authorship contribution statement)

The concept of the submission was by Dr. Juaquito Jorge MD FACS FASMBS DABOM DABS, Anila Punjwani and Gabrielle Unbehaun. Statistical analysis and Data Curation was performed by Gabrielle Unbehaun and Zachary Duarte. Anila Punjwani and Gabrielle Unbehaun wrote the first draft. Dr. Juaquito Jorge MD FACSFASMBS DABOM DABS, Anila Punjwani, Gabrielle Unbehaun, and Zachary Duarte, all reviewed, edited, and approved the final submission and publication.

## Ethical review

Authors have the ability and willingness to provide raw data in connection with a submission for editorial review, agree to not publish the same research in more than one journal or primary publication, adhere to objective reporting standards, and are willing to promptly notify the journal editor if significant errors or inaccuracies are found in published works in *Obesity Pillars.*

This submission represents original works and any work and/or words of others is appropriately cited or quoted.

Dr. Juaquito Jorge MD FACSFASMBS DABOM DABS, Anila Punjwani, Gabrielle Unbehaun, and Zachary Duarte had no involvement in the peer-review and acceptance/rejection of this submission. Responsibility for the editorial process for this article was delegated to a non-author Editor or Associate Editor.

## Source of funding

This research did not receive any specific grant from funding agencies in the public, commercial, or not-for-profit sectors.

## Declaration of competing interest

The authors declare the following financial interests/personal relationships which may be considered as potential competing interests: Affiliation with Intuitive and serves as a proctor: Dr. Juaquito Jorge MD FACS FASMBS DABOM DABS.
